# Light People: Professor Peter Delfyett

**DOI:** 10.1038/s41377-022-01065-3

**Published:** 2023-01-23

**Authors:** Hui Wang

**Affiliations:** Changchun Institute of Optics, Fine Mechanics and Physics, Chinese Academy of Sciences, 3888 Dong Nan Hu Road, Changchun, 130033 China

**Keywords:** Ultrafast photonics, Ultrafast lasers

## Abstract

In this Information Age, computers and mobile phones are important carriers of data transmission and many scientists work hard to speed up that process. Professor Peter Delfyett of the University of Central Florida has done just that. A member of the US National Academy of Engineering, he developed the world’s fastest and most powerful mode-locked semiconductor laser. I have known Professor Delfyett since 2011, when he took part in the CIOMP-OSA International Summer Session. His sincere and friendly attitude, humorous and optimistic nature and excellent academic style left a deep impression. Driven by curiosity, human beings are constantly exploring the unknown, and asking questions. Einstein once suggested, defining what the problem is can be more important than solving it. And this is what Professor Delfyett has been advocating: pursue the ultimate academic goal, ask questions, believe in yourself and never give up. In addition, he has been striving to integrate industry and scientific research, so that scientific research results can truly benefit mankind. And now, please follow Light People to the latest cutting-edge progress in optics.

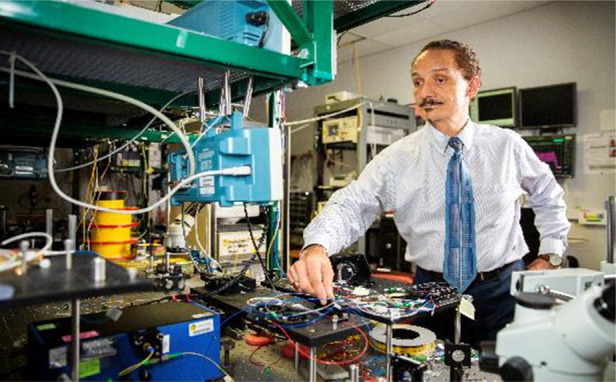

## Abstract (web summary)

Prof. Peter Delfyett has been working on semiconductor mode-locked lasers and optical frequency combs, and his research has sped up the transmission of information.

**Biography:** Peter Delfyett received his B.E. (E.E.) degree from the City College of New York (1981), M.S. degree from the University of Rochester (1983), and M. Phil and Ph.D. degrees from the Graduate School & University Center of the City University of New York (1988). After obtaining his Ph.D. degree, he joined Bell Communication Research as a member of the Technical Staff, where he concentrated his efforts towards generating ultrafast high power optical pulses from semiconductor diode lasers for applications in ultra-wide bandwidth optical signal processing and communications. In 1993, he moved to University of Central Florida. In 2003, Dr. Delfyett founded “Raydiance, Inc.” a spin-off company developing high power, ultrafast laser systems. He is a Fellow of the APS, IEEE, NAI, NSBP, Optica, and SPIE. He is also the recipient of the NSF PECASE Award, the APS Edward Bouchet Award, the 2014 Medalist from the Florida Academy of Science. Most recently, he was elected to the National Academy of Engineering for contributions to the development and commercialization of low-noise, high-power ultrafast semiconductor lasers. He has over 800 scientific publications in refereed journals and conference proceedings and 45 US patents.

**Figure:**
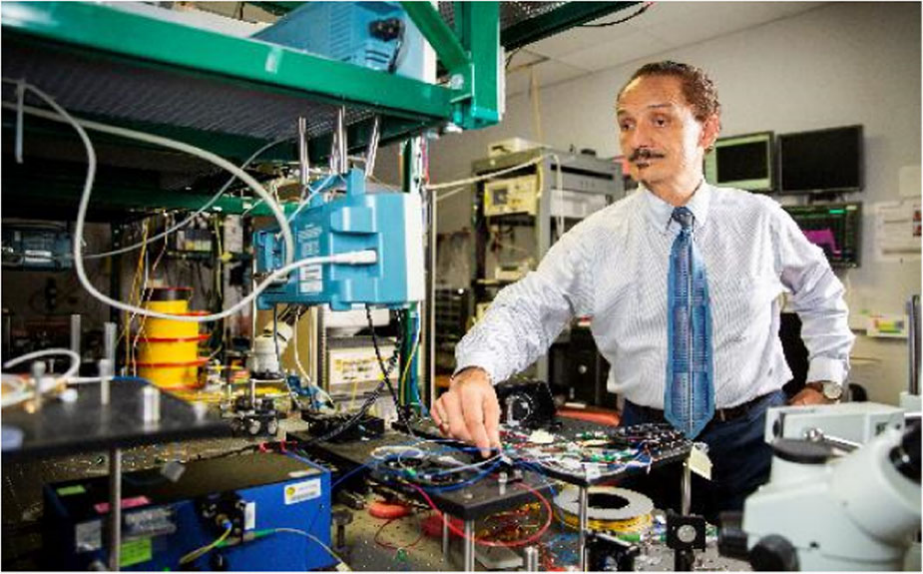

**1. Can you briefly introduce your current research direction and latest research progresses?**

Prof. Delfyett: Currently, my group is working on several projects: (1) the use of Kerr micro-combs and silicon photonics for ultra-highspeed data interconnects, (2) using injection locking techniques to synchronize Kerr micro-combs with semiconductor mode-locked diode lasers, (3) developing mode-locked diode lasers for use in nonlinear confocal microscopy, and (4) understanding and mitigating the effects of harsh environments on semiconductor mode-locked lasers (e.g., radiation environments that these devices would encounter in outer space).

**Figure:**
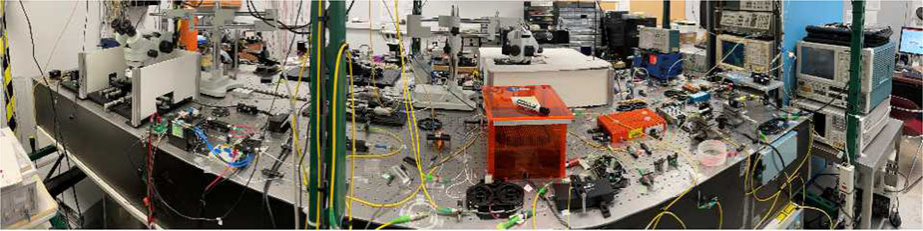
Prof. Peter Delfyett’s laboratory environment

**2. What do you think are the advantages and disadvantages of passive mode locking and soliton mode locking for integrated microring structure in semiconductor mode-locking lasers? Which one do you think is better for further increasing peak power and reducing pulse width?**

Prof. Delfyett: Passive mode-locking in semiconductor lasers using saturable absorbers is relatively easy to achieve in integrated semiconductor platform because you can tailor the saturation energy and recovery time by appropriately designing the length of the saturable absorber waveguide, and adjusting the reverse bias voltage, respectively. For shorter pulses, dispersion management would be helpful in this material platform. In addition, as pulses in semiconductor gain media get shorter, additional nonlinear effects come into play that tend to temporally broaden the pulse. This is why, for the shortest pulses and highest peak powers, the oscillating pulse in a semiconductor laser should be chirped, and use external dispersion compensation to compress the generated pulse.

In soliton mode-locking, one typically requires a balancing of dispersion and nonlinearity. More importantly, the sign of the nonlinearity must be opposite to the sign of the dispersion. So, for bright solitons, as typically encountered in fibers, the nonlinearity is positive and the dispersion is anomalous. Once this condition is met, one can generate solitons. It should be noted that the peak power, pulse width, nonlinear and dispersion are tied together, meaning that if the value of one of these parameters changes, the others must adjust accordingly. As soliton pulses get shorter and their peak powers increase, other nonlinear effects and higher order dispersion terms come into play that start to degrade the soliton pulse. These effects tend to put constraints on soliton production. In semiconductors, the nonlinearity and dispersion are positive so it is typically unfavorable for soliton production. It should be noted however, that “dark solitons” can be generated in material with normal dispersion and positive nonlinearity. (A dark soliton is a very short reduction in light intensity propagating on a continuous background of light intensity).

Regarding generating high output powers, semiconductor lasers have a very short ‘upper level’ lifetime, which makes storing energy for high output pulse powers difficult. On the other hand, if you amplify pulses that are temporally stretched, so that the stretched pulse duration is much longer than the upper state lifetime, you can circumvent this limitation. We call this “extreme chirped pulse amplification”, and we have shown the ability to generate pulses with microjoule energies—typically, semiconductors produce pulse energies of tens of picojoules. In fibers, where solitons can naturally form, the energy storage of the active ion, e.g., erbium, is orders of magnitude larger, so generating high peak power is easier.

If we consider integrated devices, the micro-ring geometries, in general, are very small, so the pulse repetition rate is high, which puts a limit on peak power. On the other hand, chip scale semiconductors have shown continuous wave output powers of several watts, operating in single transverse mode, so the potential is high. Most recently, researchers are successful in demonstrating high power, optical amplification compatible with the silicon photonics platform, so there is good opportunity for both approaches. In many instances, it will be the application that may best determine which approach or device technology is better.

**3. Semiconductor surface emitting laser (VCSEL) is attracting a lot of industry attention at the moment. How do you feel about the development of ultrafast mode-locked semiconductor laser based on VCSEL? What technical challenges does it face?**

Prof. Delfyett: In general, a VCSEL is a single longitudinal mode device, so there can be no temporal mode-locking. However, if the top mirror is removed and the structure is placed in an external cavity, we get a VECSEL (vertical external cavity semiconductor laser). These have been mode-locked and can produce very short pulses with high output power. But these devices are optically pumped, in order to maintain a high quality single transverse mode. Electrical pumping is more challenging in these devices as the large area of the gain medium allows for many transverse modes and the temporal dynamics and nonlinear effects make controlling the laser challenging. These effects lead to beam lensing, self focusing, filamentation, etc.

**4. Most semiconductor mode-locked lasers work in fundamental transverse mode. Is it possible to make a semiconductor mode-locked laser with multiple transverse modes working at the same time? What technical breakthroughs need to be made to make it happen?**

Prof. Delfyett: Transverse mode-locking dates back to the 1960’s as a way to create a scanning laser beam. Most recently, the technique has been used to create pulses with temporally varying spatial mode profiles in multimode fibers by using a combination of spatial filtering, spectral filtering, along with temporal modulation. In a semiconductor, the time scale of the dynamics, is much shorter than that in fibers and the coupling of the refractive index and carrier concentration, especially in multi-mode devices would be very dynamic, and potentially difficult to control on those time scales.

**5. You have introduced an optical frequency combs generated from electrically pumped mode-locked semiconductor lasers. Stabilized at 10** **GHz, centered at 1550** **nm, spanning 5** **THz bandwidth, it is the largest of its kind to our knowledge, directly from a semiconductor laser. Optical frequency combs have been extensively studied and there are many ways to generate them. Do you think semiconductor mode-locked lasers have any unique advantages in this direction? What are the potentials for semiconductor mode-locked lasers in the field of optical communication? What other promising applications do you think semiconductor mode-locked lasers will have in the future?**

Prof. Delfyett: Semiconductor optical frequency combs, in general, can be produced over a wide range of wavelengths, primarily determined by the material system that provides the gain. For example, you could have combs in the near UV region using GaN based materials, in the visible using GaAlAs, and in the 3 telecommunication bands (O-band, C Band and L band) using InP based materials. In communications, if you can produce a frequency comb, you can perform data and information transmission using either of the 3 basic types of communication modulation formats, for example, time domain multiplexing, wavelength domain multiplexing and code domain multiplexing.

**6. The linewidth of semiconductor lasers is an important factor affecting their performances. In recent years, researchers have achieved further narrowing of laser linewidth by injection locking using micro ring resonators or WGM resonators with high Q values. Do you think laser linewidth compression can be further improved, if so, from which likely direction?**

Prof. Delfyett: The linewidth of lasers has been dramatically been reduced recently by the coherent interaction of self injection locking with the aide of high Q resonators. Linewidths near 1 Hz have been achieved. Undoubtedly, the performance will improve as researchers better understand the underlying mechanisms. As the line widths have been reduced from typically 10’s-100’s MHz by a factor of 100 million, there is still room for improvement, as the limit to the linewidth of a laser, typically referred to as the Schawlow Townes limit, predict linewidths of ~10’s of milli-Hertz for an output power of 10’s of milliwatts. In general, it’s the output power, the losses, and stored energy that determine the linewidth, so any improvement in these areas will help.

**7. After obtaining the Ph.D. degree, you joined Bell Communication Research as a Member of the Technical Staff, how has this experience influenced your later research work?**

Prof. Delfyett: When I was a graduate student, I worked in the areas of ultrafast nonlinear laser spectroscopy, using 4-wave mixing to study phonon dynamics. At Bellcore, I had the opportunity to work in an area of developing compact laser sources of ultrashort pulses for applications in communications and signal processing, so you could say that my time at Bellcore has put me on my current research path that I am on today.

**8. You joined the School of Optics (currently, The College of Optics and Photonics) and the Center for Research and Education in Optics and Lasers (CREOL) at the University of Central Florida in 1993, why did you choose to leave industry for academia?**

Prof. Delfyett: In the early 1990’s, many of the worlds research laboratories were eliminating their ‘long term research’ directions. Research activities were primarily directed at very short goals (a few months to 1–2 years). More importantly, Bellcore was prohibited from manufacturing equipment based on the legal judgment that broke apart Bell Labs, so working in a research area that was primarily focused on developing hardware, would not be supported as a long term career path. Many of my colleagues from Bell Labs, Bellcore, etc., moved to academia to continue their research path, and I was among them.

**9. The University of Central Florida is the youngest of the three major optical research centers in the United States. What are its distinctive features compared with the University of Rochester and the University of Arizona?**

Prof. Delfyett: All three institutions are outstanding. Rochester is the oldest, Arizona is the largest, and we are the youngest. It’s an honor an privilege that we (CREOL) is considered one of the 3 optics institutions in the US.

**10. You have received numerous awards including the National Science Foundation’s Presidential Early Career Award for Scientists and Engineers (PECASE), the 2000 Black Engineer of the Year Award—Outstanding Alumnus Achievement, and the 2000 IEEE Photonics Society’s William Streifer Scientific Achievement Award, ect. Not long ago, you were also awarded the 2021 Arthur L. Schawlow Prize in Laser Science, how do you feel about these awards and honors?**

Prof. Delfyett: Being recognized by your peers is the best recognition you can have, and it’s been a tremendous honor to receive these awards. Most recently, I was elected to the National Academy of Engineering, and it is considered to be the highest honor that an engineer can achieve. It turns out that I am the first faculty at the University of Central Florida (UCF) that has been recognized with this honor for work that was done at my university with students. Since UCF is a relatively young university (~60 years old), it is truly rewarding that I could be the first at my university.

**11. As one of the very most-respected laser and photonics researchers in the world, what’s your career plan for the future?**

Prof. Delfyett: My research efforts are still in the area of communications and signal processing, but we are considering interesting applications for imaging. My plans for the future are to put diode-based frequency combs in space for communications, signal processing and imaging. The combs could also be used for precision measurements in astronomy and astrophysics.

**12. As a tutor, you have trained many students. What abilities do you most want to see in your students?**

Prof. Delfyett: The most important attribute I like to see in students are a desire to continue to learn and a strong work ethic. Research is process that takes a long time to achieve results and if your personality likes to achieve results on a short time scale, research may not be best suited for those individuals. For me, I recognize research is a “delayed gratification” activity, so I get my short-term or “instantaneous gratification” from teaching. If I teach a really good lecture, my students let me know right away. This makes the long periods of time between research breakthrough tolerable.

**Figure:**
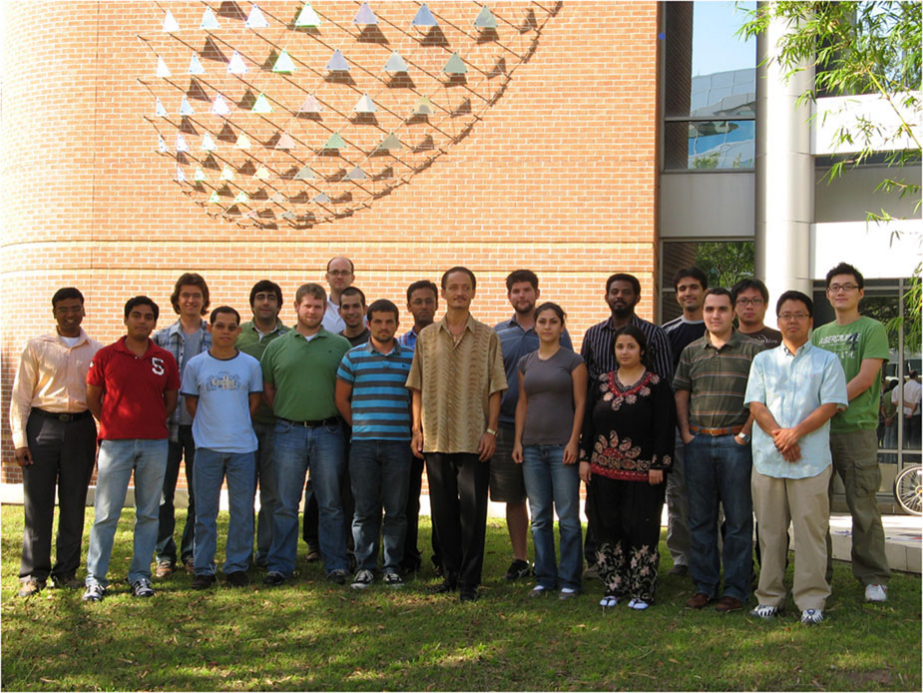
Prof. Peter Delfyett and his team members

**13. You took part in the CIOMP-OSA International Summer Session in 2011 at CIOMP. Is it the first time you visit CIOMP? What was your impression?**

Prof. Delfyett: My visit to CIOMP in Changchun was great. There were many enthusiastic students, and I was honored to be able to share my research and passion with these students. The International Summer Session that we had there has gone on to become the Siegman Summer School, in honor of Prof. Anthony E. Siegman.

**Figure:**
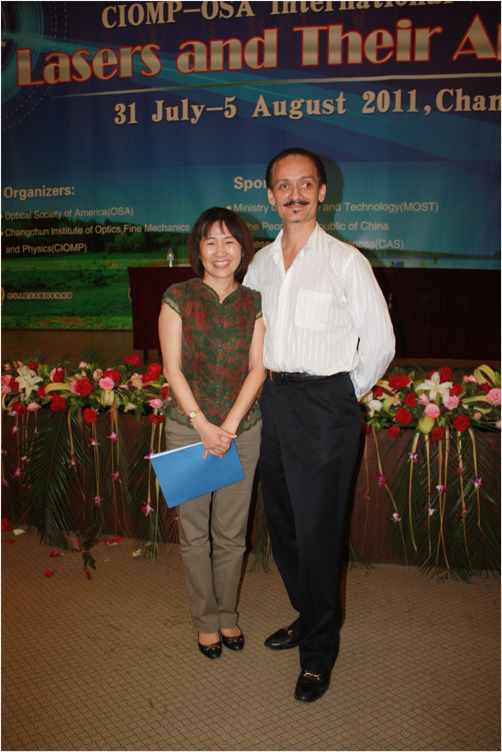
Prof. Peter Delfyett with the Director of the current Light Publishing Group Dr. Yuhong Bai

**Figure:**
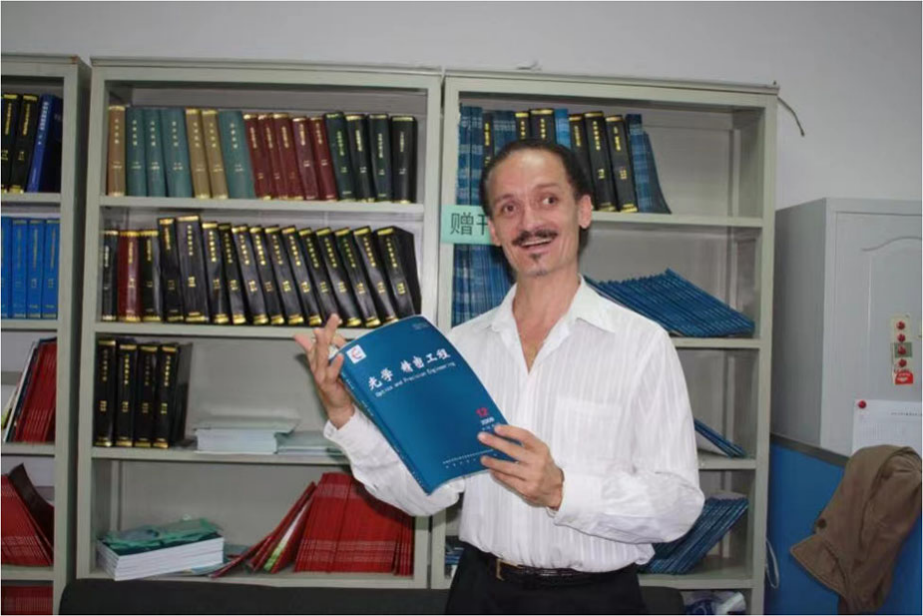
Prof. Peter Delfyett visiting the Editorial Office in CIOMP

**Figure:**
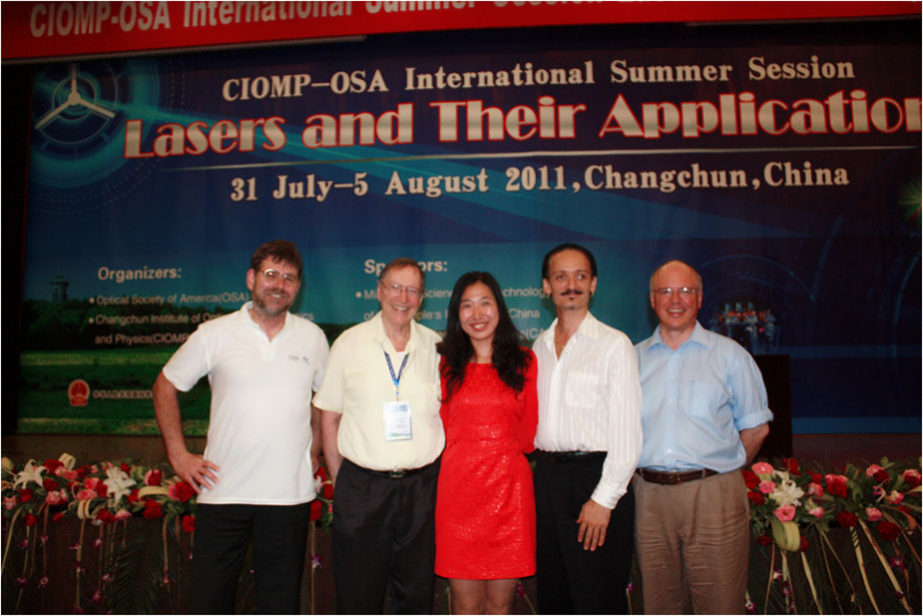
Group photo (from left to right: Peter Unger, James Harris, Hui Wang, Peter Delfyett, David Miller)

**Figure:**
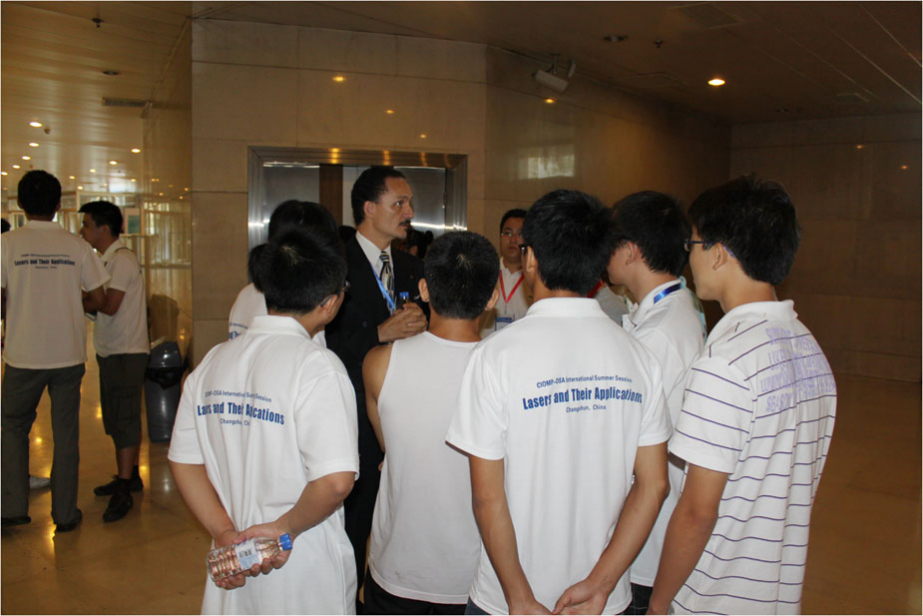
Prof. Peter Delfyett talking with the students who participating the CIOMP-OSA International Summer Session in 2011

**14. You said once, “For me, excellent academic means always shooting for the stars. Always do your best and always question.” What in your opinion counts as doing one’s best in scientific research? Would you feel annoyed by a student or young staff who always asks you questions?**

Prof. Delfyett: Doing your best in research is not stopping at achieving a good result. It’s important to continue to ask questions, and to continue to push the boundaries of what we know and what we can do. I would never get annoyed by anyone that likes to ask questions because this shows that they have the desire to continue to learn and be better.

**15. What do you think should be the relationship between scientific research results and their commercialization?**

Prof. Delfyett: I believe that there are two types of research —(1) curiosity driven research, where the application of the new knowledge may not be immediately recognizable, and (2) application driven research, where the work you are doing can have an impact on pushing the state of the art of technology. I believe that you can do both… However, in today’s global society, researchers must apply for grants to raise the funding to support students and purchase the materials and supplies needed for the work. In general, since the funding comes from either government or industrial sources, there is a desire to know what the ‘return on investment’ could be. Therefore, I think it is important for researchers to seriously consider how to tailor, or modify, their work so that the potential for commercialization can be realized.

**16. I read that when you first fell in love with science, you wanted to be a paleontologist. But eventually you chose optics and photonics instead. Why made you change your mind? What kind of career do you think you would have if you haven’t become a scientist?**

Prof. Delfyett: I wanted to be a paleontologist when I was in first grade (about the age of 7). Once I got to college, I recognized that engineering would be a better choice as getting a job as a paleontologist could be extremely difficult. As a young electrical engineering student, I was looking at the course catalog one evening, as I was trying to plan out which elective course I could take, and I saw the course “Introduction to Lasers and Masers”. IN the course description, it said, “This course will also cover the basics of fiber optic communications”. This was about the year 1978 or so. Fiber optics was a really hot topic and I concluded that if I pursue this field, since it was so new, it could possibly enable me to do this for an entire career. Other areas that I considered were music, and finance/economics. I had also considered biomedical engineering/robotics as well.

**17. What is the biggest turning point in your career?**

Prof. Delfyett: I would say that the biggest turning point in my career was going to Bell Communications Research (Bellcore), because it was there that I stared researching ultrafast diode lasers.

**18. In your career, has anyone had a major influence on you? In what way?**

Prof. Delfyett: My research advisor was a big influence on me. He was the person that is most responsible for me pursing ultrafast optical science and engineering. Many of my research habits are attribute able to him, such as being able to dig deep into a problem, but not getting lost in the weeds, and to always ‘look up’ and see where you are going as you dig deep into a problem, to make sure you’re still going in the right direction.

**Figure:**
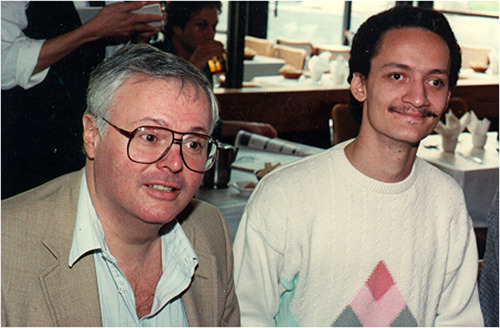
Prof. Peter Delfyett in his studenthood with his PhD advisor Prof. Robert. R. Alfano

**19. How do you balance your work and family life?**

Prof. Delfyett: Balance is very important. I try to do things, such as hobbies, that require me to completely disconnect from science. Exercising is also important, as it keeps the body healthy and that, in turn, helps keep the mind healthy, too.

**20. What are your hobbies?**

Prof. Delfyett: I have several hobbies, but the 2 that I mainly focus on are (1) playing drums, and (2) dancing salsa. I like both because they require you to be completely mentally engaged with what you are doing. This allows my mind to disengage from science, so that when I start to re-think about science, my mind is fresh and can think about new possible solutions or directions to take.

**Figure:**
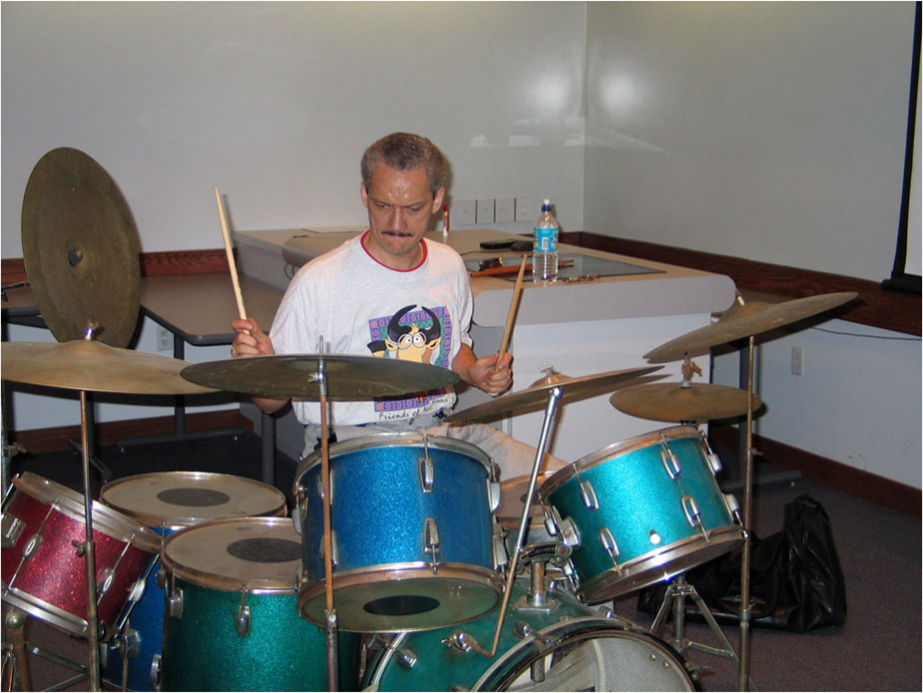
Prof. Peter Delfyett playing the drums

**Figure:**
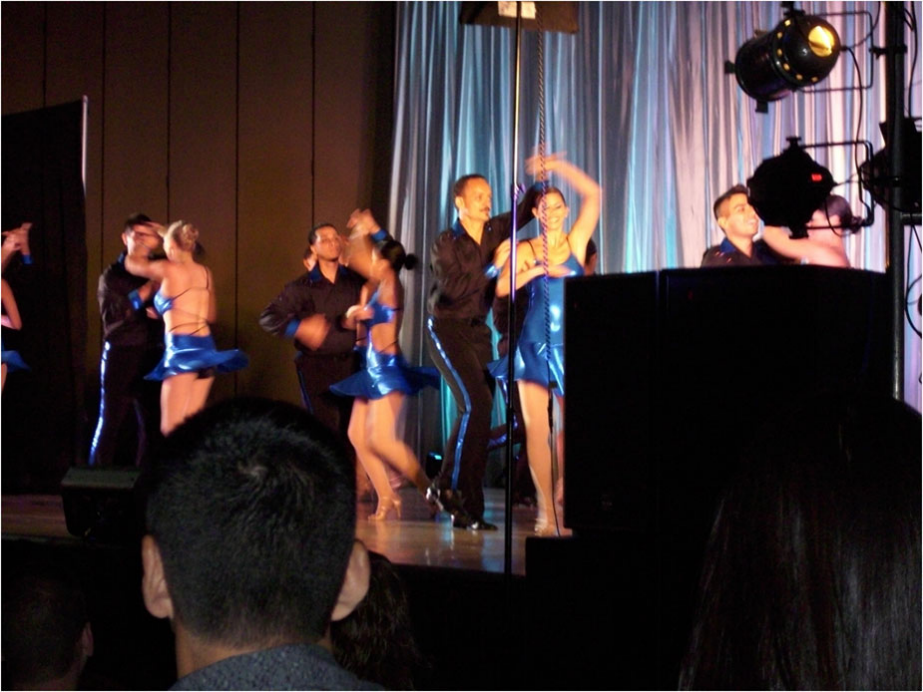
Prof. Peter Delfyett dancing “salsa” with a performance team

**21. What advice and suggestions would you give our young audience on life and career?**

Prof. Delfyett: I think my best advise for young people is to (1) believe in yourself—you can to it, because if you say “no, I can’t” it is certain that you won’t. (2) Never give up—persistence is probably the most important aspect of doing anything. Greatness doesn’t happen just because you show up on the first day. (3) Balance—it is something as necessary as food, air and water. If there is no balance, the imbalance may not allow you to see what is possible and (4) do what you enjoy and what you’re good at—when you can do this, you really never work a day in your life.

